# A rapid lateral flow immunoassay strip for detection of SARS‐CoV‐2 antigen using latex microspheres

**DOI:** 10.1002/jcla.24091

**Published:** 2021-11-06

**Authors:** Lin Shen, Qihan Zhang, Xiaolu Luo, Haolin Xiao, Miao Gu, Liangli Cao, Feijun Zhao, Zhencheng Chen

**Affiliations:** ^1^ School of Life and Environmental Sciences Guilin University of Electronic Technology Guilin China; ^2^ School of Electronic Engineering and Automation Guilin University of Electronic Technology Guilin China; ^3^ Clinical Laboratory The Fourth People's Hospital of Nanning Nanning China; ^4^ School of Materials Science and Engineering Guilin University of Electronic Technology Guilin China

**Keywords:** EDC and NHS, latex microspheres, SARS‐CoV‐2 antigen

## Abstract

**Background:**

Severe acute respiratory syndrome coronavirus 2 (SARS‐CoV‐2) is a highly infectious and concealed virus that causes pneumonia, severe acute respiratory syndrome, and even death. Although the epidemic has been controlled since the development of vaccines and quarantine measures, many people are still infected, particularly in third‐world countries. Several methods have been developed for detection of SARS‐CoV‐2, but owing to its price and efficiency, the immune strip could be a better method for the third‐world countries.

**Methods:**

In this study, two antibodies were linked to latex microspheres, using 1‐(3‐dimethylaminopropyl)‐3‐ethylcarbodiimide hydrochloride and N‐hydroxysuccinimide, as the bridge to decrease the cost further and improve the detection performance. The specificity of the lateral flow immunoassay strip (LFIA) was tested by several common viruses and respiratory bacterial infections. Besides, the reproducibility and stability of the LFIAs were tested on the same batch of test strips. Under optimal conditions, the sensitivity of LFIA was determined by testing different dilutions of the positive specimens.

**Results:**

The proposed LFIAs were highly specific, and the limit of detection was as low as 25 ng/mL for SARS‐CoV‐2 antigens. The clinical applicability was evaluated with 659 samples (230 positive and 429 negative samples) by using both LFIA and rRT‐PCR. Youden’s index (J) was used to assess the performance of these diagnostic tests. The sensitivity and specificity were 98.22% and 97.93%, respectively, and J is 0.9615. The sensitivity and specificity were 98.22% and 97.93%, respectively, and J is 0.9615. In addition, the consistency of our proposed LFIA was analyzed using Cohen's kappa coefficient (κ = 0.9620).

**Conclusion:**

We found disease stage, age, gender, and clinical manifestations have only a slight influence on the diagnosis. Therefore, the lateral flow immunoassay SARS‐CoV‐2 antigen test strip is suitable for point‐of‐care detection and provides a great application for SARS‐CoV‐2 epidemic control in the third‐world countries.

## Graphical abstract

Severe acute respiratory syndrome coronavirus 2 (SARS‐CoV‐2) is a highly infectious and concealed virus that causes pneumonia, severe acute respiratory syndrome, and even death. Although the epidemic has been controlled since the development of vaccines and quarantine measures, many people are still infected, particularly in third‐world countries. Several methods have been developed for detection of SARS‐CoV‐2, but owing to its price and efficiency, the immune strip could be a better method for the third‐world countries. However, some disadvantages of the strip can be fixed to decrease the cost and improve the performance. In this study, two antibodies were linked to latex microspheres, using 1‐(3‐dimethylaminopropyl)‐3‐ethylcarbodiimide hydrochloride and N‐hydroxysuccinimide, as the bridge to decrease the cost further and improve the detection performance. Unlike the reverse‐transcriptase‐polymerase chain reaction (rRT‐PCR) test, the results showed that the proposed strip had high specificity for SARS‐CoV‐2 antigen, and no cross‐reactions with other common viruses was observed. Most importantly, the detection limit was as low as 25 ng/ml, and the cost decreased to $0.15 per strip. Thus, the proposed SARS‐CoV‐2 antigen test strip offers a cheap, rapid, sensitive, specific, and visual method for SARS‐CoV‐2 antigen detection and shows great potential for COVID‐19 epidemic control in third‐world countries.

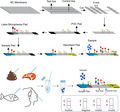

## 1 INTRODUCTION

In December 2019, a new coronavirus, SARS‐CoV‐2 was identified as the cause of an ongoing pandemic that originated in Wuhan, China.^1^, ^2^, ^3^, ^4^ In February 2020, the disease was officially named coronavirus disease 2019 (COVID‐19) by the World Health Organization.^5^, ^6^, ^7^ As of July 7, 2021, >185.36 million cases have been reported across 188 countries, and >30.66 million cases have been reported in India. A better way to control or stop the spread of outbreaks is to perform high‐quality and high‐frequency representative sampling for serological testing because quality and frequency are far more significant than the assay's sensitivity.

The rRT‐PCR test is considered the ‘gold standard’ for the qualitative detection of nucleic acid from SARS‐CoV‐2 found in respiratory specimens, which is characterized by high sensitivity, rapid detection, and specificity.^8^, ^9^, ^10^, ^11^ However, three crucial issues with the rRT‐PCR test hinder the prevention of the epidemic: cost, testing time, and testing frequency. Due to these issues, colloidal gold immunochromatography of SARS‐CoV‐2 IgG and IgM antibody detection was attempted to slow down the spread of the epidemic.^12^ Although it is simple, easy, and suitable for on‐site screening, the generation of virus‐specific antibodies requires a long “window period” of 10–28 days.^13^, ^14^ During this period, SARS‐CoV‐2 continues to spread. Hence, a new testing strategy should be developed to detect SARS‐CoV‐2 which is rapid, inexpensive, sensitive, and exhibits stable characteristics.

Nucleocapsid protein (N‐protein) could be an alternative biomarker to detect one of the COVID‐19 antigens^15^ because it not only creates the capsid to enclose the nucleic acid, but also interacts with the viral membrane protein during viral assembly, and is even the most abundant protein in coronavirus.^16^, ^17^ In addition, COVID‐19 antigens can be collected in multiple ways, such as nasopharyngeal and oropharyngeal swabs, sputum, meat, or even feces. Recently, latex microspheres have gained significant attention as a more sensitive label for lateral flow immunoassay strips (LFIA) because the traditional label of colloidal gold cannot meet the detection limit demand.^18^, ^19^, ^20^

Hence, in the present study, we developed a rapid immunoassay strip targeting the detection of SARS‐CoV‐2 antigens. Latex microspheres were used to label the antigens to enhance the detection limit. Additionally, we tested 659 samples by both the proposed immunoassay and the rRT‐PCR and hoped to provide a supplementary point‐of‐care diagnostic detection approach to control the spread of COVID‐19.

## 2 METHODS

### 2.1 Materials

We purchased potassium chloride (KCl, 99%), dipotassium hydrogen phosphate (K_2_HPO_4_, 99%), disodium hydrogen phosphate (Na_2_HPO_4_, 99%), monosodium phosphate (NaH_2_PO_4_, 99%), and sodium chloride (NaCl, 99%) from Sinopharm Chemical Reagent Co., Ltd., while Bovine serum albumin (BSA) was from Shanghai Roche Pharmaceuticals Co., Ltd.. In addition, hydrochloric acid (HCl, 37%), tween‐20 (40%), and 2‐(N‐Morpholino) ethanesulfonicacid (MES, 99%) were purchased from Sigma‐Aldrich (Shanghai) Co., Ltd. Recombinant mouse anti‐human novel coronavirus first antibody and recombinant mouse anti‐human novel coronavirus second antibody were purchased from Beijing Yiqiao Shenzhou Science and Tech Co., Ltd. The 2019‐nCoV recombinant N‐protein and goat anti‐mouse IgG were purchased from Shenzhen Crystalo Biopharma Tech Co., Ltd.. Latex microspheres were purchased from Shanghai So‐Fe Biomedicine Tech Co., Ltd., and nitrocellulose membrane, gold colloidal conjugate pads, whole blood separation membrane, adhesive PVC backing, absorbent pads, plastic card waterproof pads, and sample pads were from Shanghai Jieyi Biotechnology Co., Ltd. D‐ (+)‐trehalose dihydrate was purchased from TCI Chemical Industry Development Co., Ltd. 1‐(3‐Dimethylaminopropyl)‐3‐ethylcarbodiimide hydrochloride (EDC) and N‐hydroxysuccinimide (NHS) were purchased from Thermo Fisher Scientific. 2‐Amino‐2‐(hydroxymethyl)‐1,3‐propanediol (Tris) was purchased from VWR International China Co., Ltd. The study was approved by the Ethics Committee of Guilin University of Electronic Technology (No: GUETIEC‐2020001). All samples were collected and tested at The Fourth People's Hospital of Nanning, Chongqing University Three Gorges Hospital, and Chongqing Public Health Medical Treatment Center. All the samples were leftover samples, and informed consent process was not required for this type of samples according to institutional review board approval. All the clinical diagnostic criteria for each disease were followed the Chinese‐related guidelines.

### 2.2 Instruments

All buffers and solutions were prepared using ultrapure water purified by a Milli‐Q purification system (Millipore). MES cold buffer was prepared using a pH meter (Shanghai INESA Scientific Instrument Co., Ltd.,). The latex microspheres were centrifuged in a frozen centrifuge (Shanghai Anting Scientific Instrument Factory,) and were incubated in a rotary culture apparatus (Haimen Kylin‐Bell Lab Instruments Co., Ltd.,). The centrifuged latex microspheres were subjected to ultrasound in a JY99‐IIDN ultrasonic cell crusher (Ningbo Xinzhi Biotechnology Co., Ltd.,). The latex microsphere pads and antibody‐coated were dried in an air‐blast drying oven (Shanghai Jing Hong Laboratory Instrument Co., Ltd.,). The recombinant mouse anti‐human novel coronavirus secondary antibody and goat anti‐mouse IgG were coated using the BioDot‐XYZ3210 three‐dimensional spraying platform (Shanghai Kinbio Tech Co., Ltd.,). The test strips were cut using a BioDot‐CM4000 cutting machine (Shanghai Kinbio Tech Co., Ltd.,).

### 2.3 Preparation of solutions

Sodium chloride (0.09 g) and tween‐20 solution (0.05 ml) were added to ultrapure water, and the volume was set to 100 ml as the sample diluent. D‐(+)‐Trehalose Dihydrate (0.03 g) and phosphate buffer 1 ml (0.1 mol/L) were added to ultrapure water (9 ml) as dilution liquid. MES of 0.976 g (195.23 g/mol) was added to ultrapure water and set the volume at 50 ml as MES cold buffer (100 mM). NHS (10 mg) and EDC (10 mg) were dissolved in MES cold buffer (500 μl). Tween‐20 solution (0.15 ml) was added to BSA solution (48 ml) drop by drop, and the pH value was set to 8.0, and the volume was set to 60 ml as reconstituted fluid.

### 2.4 Preparation of functional Latex microspheres with protein molecules

The latex microspheres (25 μl) were diluted with MES cold buffer (1 ml), then the buffer was centrifuged at 12000 rpm for 15 min, and the supernatant liquid was discarded twice. Next, NHS (10 μl) and EDC (5 μl) solutions were added to the mixture solution and incubated on a blood mixer at room temperature for 20 min. Then, the mixture was centrifuged and washed at 12,000 rpm for 15 min twice. Finally, recombinant mouse anti‐human novel coronavirus first antibody (100 μl) was added to the mixture solution, and the volume was set to 1 ml, on a blood mixer for 2 h at room temperature.

### 2.5 Preparation of reaction area

The latex microspheres were centrifuged at 12,000 rpm for 15 min and washed twice with 1 ml of MES cold buffer. Then, the volume of latex microspheres was set to 1 ml with 10% BSA solution and incubated on a blood mixer for 1 h. Finally, the latex microspheres solution was dropped on a pad and heated at 37°C for 6 h for later use.

The recombinant mouse anti‐human novel coronavirus second (2.5 mg/ml) antibody and goat anti‐mouse IgG (2 mg/ml) were diluted. The nitrocellulose membrane was then attached to the corresponding position of the PVC bottom plate. The test line (1 mm) was drawn using a three‐dimensional spraying platform (BioDot‐XYZ3210) on a nitrocellulose membrane at a speed of 1 μl/cm. The control line was drawn with the same width (1 mm) and speed (1 μl/cm). The nitrocellulose membrane coated with antibody was heated at 37°C for 4–6 h for later use.

PBS buffer (5 ml) was added to ultrapure water (800 ml), and then tween‐20 solution (5 g) was added to the solution, and the volume was set to 1000 ml. The sample pads were soaked in the solution for 3 min and heated at 55°C for 4 h for later use.

### 2.6 Fabrication of lateral flow immunoassay strip

**FIGURE 1:**
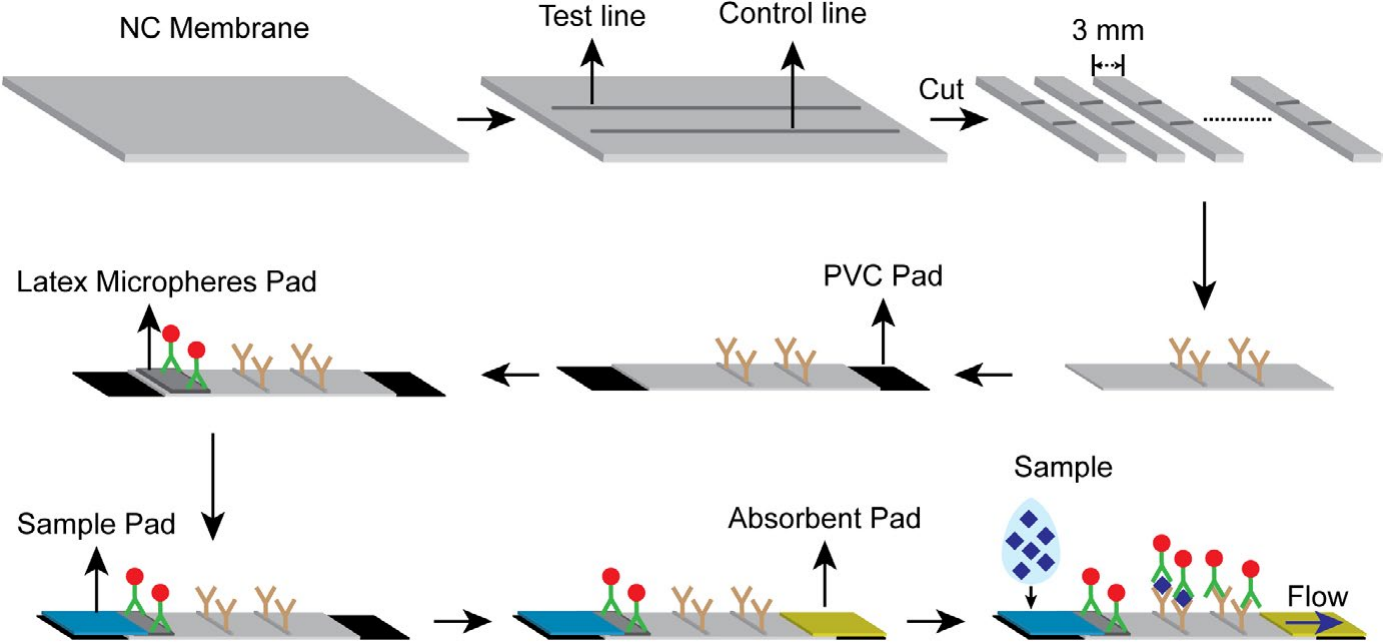
Fabrication of immunoassay strip and detection principle

The stepwise fabrication process is illustrated in Figure 1. Specifically, the nitrocellulose membrane pad was attached to the PVC pad first. Then, the latex microsphere, sample, and absorbent pads were sequentially attached. The strip was cut to a 3 mm width and fabricated in a commonly used shell.

### 2.7 Detection procedure and statistical analysis

**FIGURE 2:**
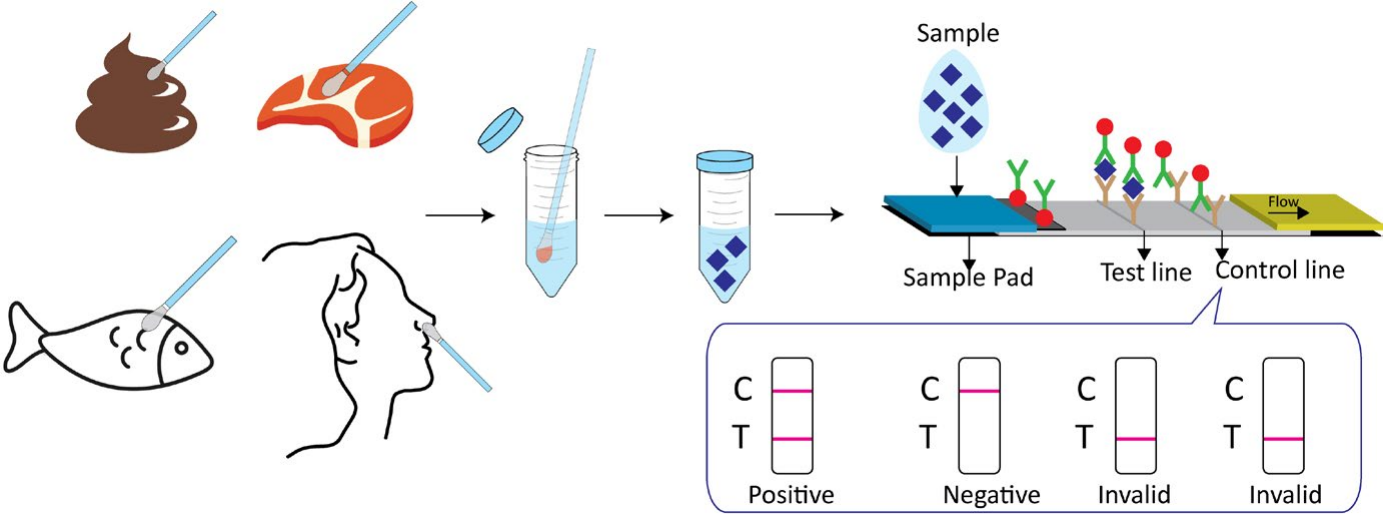
The simple detection procedure of proposed LFIA and test results

The detection procedure was performed as follows: as shown in Figure 2, if the sample was refrigerated or frozen for storage, the sample and the required reagent were removed from the storage condition until it reached room temperature (15–30°C). After the sample and reagent were at room temperature, the sample was shaken well before testing. Next, the immunoassay strip was removed and placed on a flat horizontal desktop. The collected swab sample from the deep pharynx, meat, or feces was then placed into a sample diluent with stirring five times and immersed for 1 min. Next, the swab was broken and shook five times. Subsequently, two drops of the diluted solution were added into the test hole of the immunoassay strip to ensure that there was no bubble during the operation. Finally, the results were read and recorded within 10 min.

## 3 RESULT AND DISCUSSION

### 3.1 Size of Latex microspheres

**FIGURE 3:**
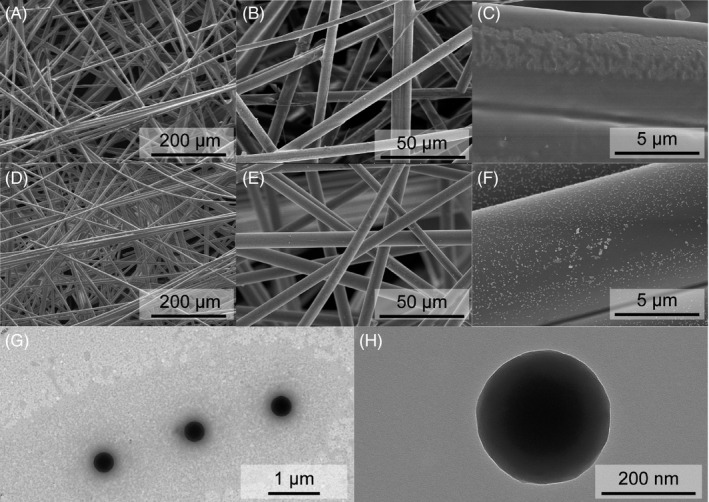
SEM images of (A‐C) different magnification ration of bare NC membrane, (D–F) different magnification ration of NC membrane modified with latex microspheres, and (G‐H) TEM images of latex microsphere

The antigens, antibodies, and latex microspheres were reacted on the NC membrane. Scanning electron microscope (SEM) and transmission electron microscopy (TEM) were employed to investigate the morphology and size of the latex microspheres. Figure 3A–C) shows the bare NC membrane at different magnifications of 200 μm, 50 μm, and 5 μm. The pore size was 7.5 μm and nothing was observed on the pore surface. As shown in Figure 3D,F, the pore size was still 7.5 μm and nothing could be observed at magnifications of 200 μm and 50 μm, but latex microspheres that were evenly spread on the surface, were clearly observed. However, the magnification of 5 μm was much larger than the size of the latex microspheres. Subsequently, TEM was employed to investigate the size of the latex microspheres. As shown in Figure 3G, the latex microspheres were evenly dispersed on the surface, and the diameter of each latex microspheres was 200 nm, indicating that the strip could sustain a stable performance.

### 3.2 LFIA optimization

**FIGURE 4:**
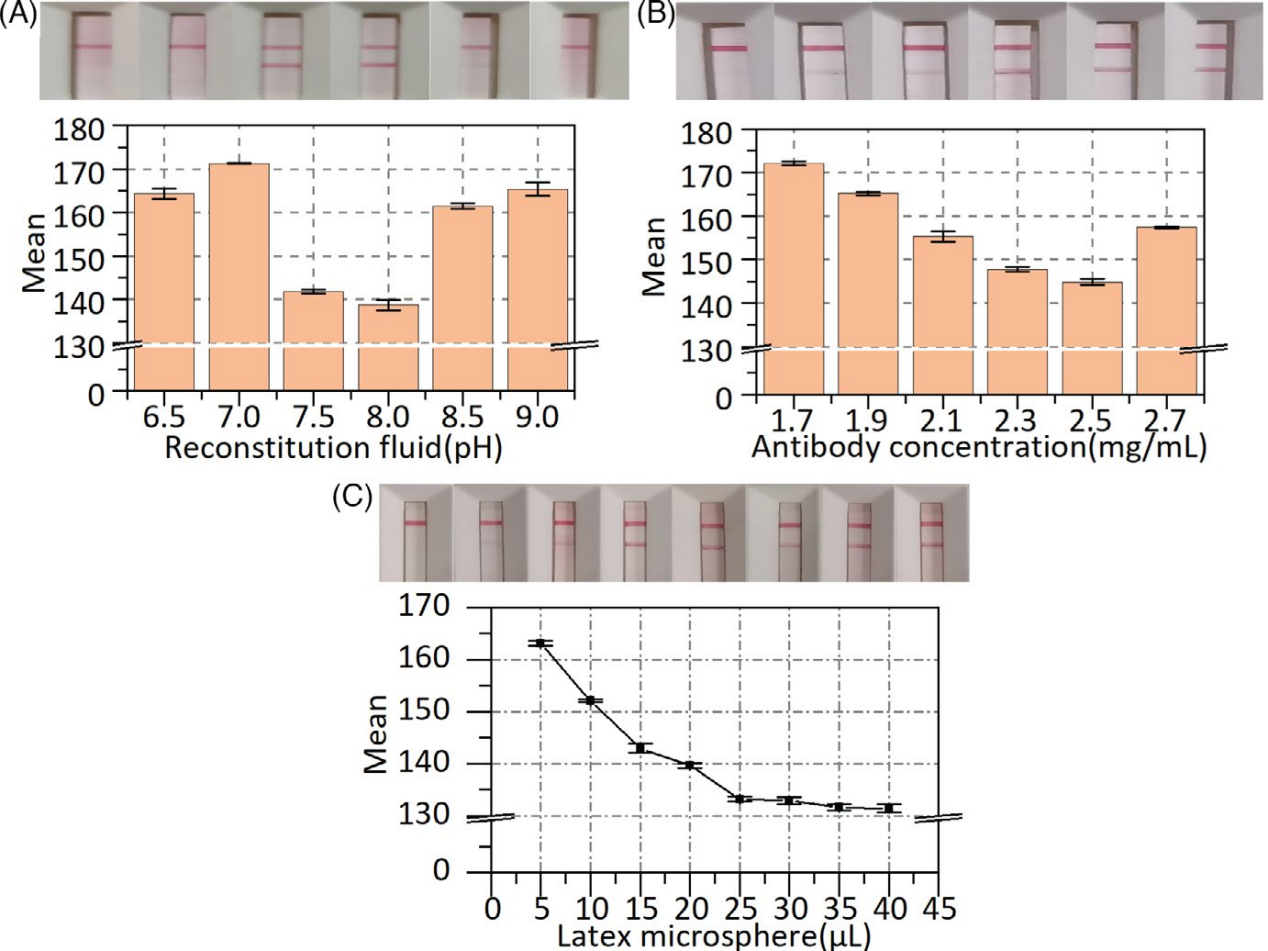
LFIA optimization. (A) Optimization of pH value of the reconstitution fluid. (B) Optimization of antibody concentration on test line. (C) Optimization of latex microsphere. Error bar represents the standard deviation of three repetitive experiments

Several of factors have been explored to improve the performance of LFIA. Different concentrations of the recombinant mouse anti‐human novel coronavirus second antibody on the T line were optimized. The color intensity of the test strips was measured using the mean gray value of ImageJ. As shown in Figure 4B, for the positive sample, the mean gray value of the T line decreased to the minimum and then increased when the recombinant mouse anti‐human novel coronavirus second antibody concentration of 2.5 mg/ml was used as the optimal concentration. Similarly, the pH of the reconstitution fluid was also investigated. When the pH of the reconstitution fluid was 8, as shown in Figure 4A, the mean gray value of the T line reached its lowest value. Hence, the pH of 8 was considered as the optimal reconstitution fluid pH value. The influence of latex microspheres on LFIA was also explored. As shown in Figure 4C, the mean gray value of the T line decreased with increasing concentrations of the latex microspheres. Meanwhile, the mean gray value of the T line tended to be smooth when the latex microspheres exceeded 25 μl. Hence, a 25 μl volume of latex microspheres was selected as optimal.

### 3.3 Specificity, reproducibility, stability, and sensitivity of LFIA

**FIGURE 5:**
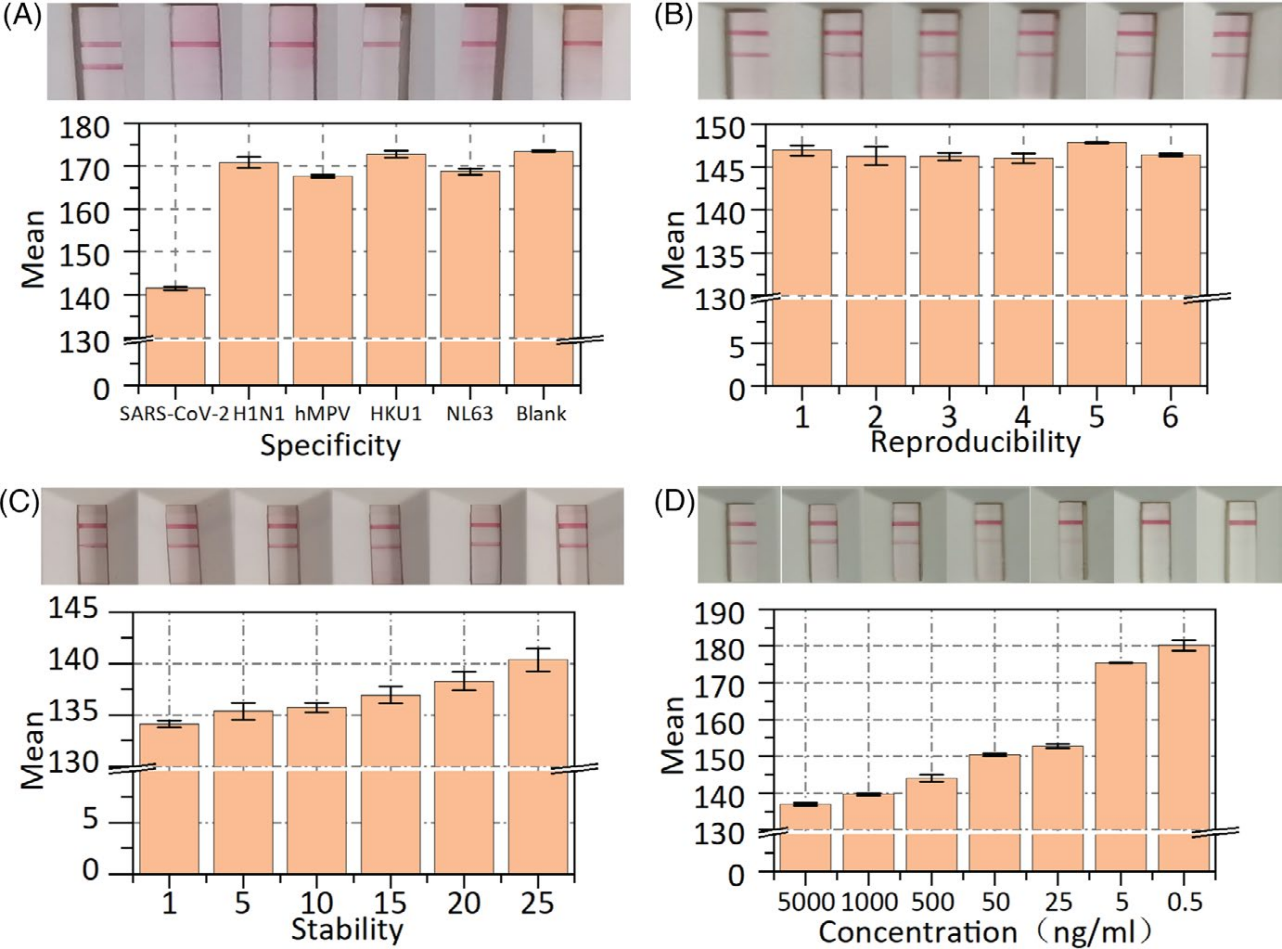
The specificity, reproducibility, stability, and sensitivity analysis of the LFIA. (A) Mean gray value and test results of SARS‐CoV‐2 antigen, H1N1, hMPV, HKU1, NL63 and blank control. (B) Test results and mean gray value of reproducibility on the same batch of six strips. (C)Test results and mean gray value of stability on the same batch of six strips. (D) Test results and mean gray value of the sensitivity of the LFIA. Error bar represents the standard deviation of three repetitive experiments

To evaluate the specificity of LFIA, we detected several common viruses and respiratory bacterial infections, such as influenza virus (H1N1), human metapneumovirus (hMPV), coronavirus (HKU1), and coronavirus (NL63). The common viruses (75 μl) with the highest clinical concentration were added individually to the sample solutions. The sample solutions without adding any analyte were used as the blank groups and the inactivated SARS‐CoV‐2 antigen for the positive group. A pipette was used to add the sample to the test strip (3 drops, ~100 μl), which was then placed on a horizontal surface for 10 min before examining the test strip for bands. As shown in Figure 5A, the mean gray value from the LFIA for SARS‐CoV‐2 antigen declined to low levels, while the mean gray values for other common viruses were close to the blank values. Moreover, we added some common viruses (H1N1, hMPV, HKU1, NL63, etc.) to two SARS‐CoV‐2 antigen‐positive specimens (PS‐1, PS‐2) and two SARS‐CoV‐2 antigen‐negative specimens (NS‐1, NS‐2). Before and after adding the interferences, we tested the same batch of test strips. As shown in Table S1, the proposed LFIA still showed the same results after adding the interferences, indicating that the proposed LFIA does not cross‐react with the above similar viruses or bacterial antibodies. The results in Figure 5A and Table S1 indicate that the test strips have good specificity and anti‐interference performance. In this study, the tests were repeated at least three times.

**TABLE 1:** The results of LFIA and rRT‐PCR

Test results	rRT‐PCR results		Total
	Positive	Negative	
Positive	221	4	225
Negative	9	425	434
Total	230	429	659

The reproducibility of the LFIA was tested using the same weak‐positive specimens on the same batch of test strips. Six test strips were present in every group, and each batch of test strips was tested in at least five groups. As shown in Figure 5B, the T line was easily observed, and the mean gray value of the corresponding T line reached approximate levels, indicating that the proposed test strip has good reproducibility. In addition, the stability of the LFIA was tested within one month with the same positive specimen. After each use, the positive specimen was placed in a refrigerator at 4°C. The LFIA was tested every five days, with a minimum of five test strips for each test. As shown in Figure 5C, the results show that the mean gray value of the test line increased linearly because some antibodies gradually became inactivated over time. However, the results were very sensitive to the mean gray value, but the color of the test line was very difficult for the human eye to distinguish, demonstrating that the proposed LFIA has great stability.

Under optimal conditions, the sensitivity of LFIA was determined by testing different dilutions of the positive specimens. As shown in Figure 5D, the mean gray value of the T line increased with decreasing concentration of the specimens. When the concentration of the specimen was below 25 ng/ml, the mean gray value of the T line reached the highest value. Consequently, in our lateral flow immunoassay strip, the limit of detection was as low as 25 ng/ml.

Besides, the meat or faces samples could be tested by our proposed LFIA. But the positive sample cannot be found, so we just tested the negative samples. 20 negative samples (10 meat samples and 10 faces samples) were tested. As we expected, the test strip test results all showed negative and each sample was repeated at least three times. Inactivated samples were used for the specificity, reproducibility, stability, and sensitivity tests. According to the Chinese‐related guidelines, these tests were operated in a BSL II laboratory in Guilin University of Electronic Technology.

### 3.4 Comparative studies between LFIA and rRT‐PCR

A total of 659 subjects from three hospitals were enrolled in this study from January 2020 to December 2020. To evaluate the clinical applicability of the proposed LFIA, 659 samples (230 positive and 429 negative samples) were tested using LFIA and rRT‐PCR tests at the same time in the Fourth People's Hospital of Nanning, Chongqing University Three Gorges Hospital, and Chongqing Public Health Medical Treatment Center. As shown in Table 1, only four samples were tested as false positives, and nine samples were tested as false negatives. Youden's index (J) was used to assess the performance of these diagnostic tests. The sensitivity and specificity were 98.22% and 97.93%, respectively, and J is 0.9615. In addition, the consistency of our proposed LFIA was analyzed using Cohen's kappa coefficient (κ = 0.9620). Besides, 79 positive samples were collected from mild patients, 101 positive samples were collected from moderate patients, and 50 positive samples were collected from severe patients. As shown in Table S5, disease stage, age, gender, and clinical manifestations have only a slight influence on the diagnosis. These results exhibit not only great diagnostic performance but also remarkable diagnostic consistency. Hence, the proposed LFIA should be a feasible and perfect supplement diagnostic test for detecting SARS‐CoV‐2 in clinical laboratories or even point‐of‐care testing.

## 4 CONCLUSION

In this study, we developed a lateral flow immunoassay strip using NHS‐EDC and latex microspheres for the rapid and visual detection of the SARS‐CoV‐2 antigen. Under the optimal assay conditions, only 300 μl of sample diluent was required to detect the SARS‐CoV‐2 antigen within 3 min. The proposed LFIAs were highly specific, and the limit of detection was as low as 25 ng/ml for SARS‐CoV‐2 antigens. The results exhibited comparable accuracy and reproductivity compared with rRT‐PCR‐based test. In addition, the proposed LFIA was rapid, easy to use and costed only $0.15 per test. Therefore, the lateral flow immunoassay SARS‐CoV‐2 antigen test strip is suitable for point‐of‐care detection and provides a great application for SARS‐CoV‐2 epidemic control in the third‐world countries.

## Supporting information

Table S1‐S5

Supplementary Material

## Data Availability

The data that support the findings of this study are available from the corresponding author upon reasonable request.
