# Supplementary material for: A rapid lateral flow immunoassay strip for detection of SARS‐CoV‐2 antigen using latex microspheres
Source: J Clin Lab Anal. 2021 Nov 6;35(12):e24091. doi: 10.1002/jcla.24091 (PMC8646881; doi:10.1002/jcla.24091)
Supplement: Supplementary file 1 — Table S1‐S5 [file JCLA-35-e24091-s001.docx]

**Electronic supplementary material (ESI)**

We added some common viruses (H1N1, hMPV, HKU1, NL63, and etc.) to two SARS-CoV-2 antigen-positive specimens (PS-1, PS-2) and two SARS-CoV-2 antigen-negative specimens (NS-1, NS-2). Before and after adding the interferences, we tested the same batch of test strips. As shown in Table S1; the proposed LFIA still showed the same results after adding the interferences, indicating that the proposed LFIA does not cross-react with the above similar viruses or bacterial antibodies. ‘+’ stands for positive, and ‘-‘ stands for negative.

Table S1 Analytical specificity and anti-interference performance of other viruses

| Specimen No. | Pc1 | | Pc2 | | N1 | | N2 | |
| --- | --- | --- | --- | --- | --- | --- | --- | --- |
|  | Before  Adding | After  adding | Before  adding | After  adding | Before  adding | After  adding | Before  adding | After  adding |
| H1N1-1 | + | + | + | + | - | - | - | - |
| H1N1-2 | + | + | + | + | - | - | - | - |
| H1N1-3 | + | + | + | + | - | - | - | - |
| H1N1-4 | + | + | + | + | - | - | - | - |
| H1N1-5 | + | + | + | + | - | - | - | - |
| H3N2-1 | + | + | + | + | - | - | - | - |
| H3N2-2 | + | + | + | + | - | - | - | - |
| H3N2-3 | + | + | + | + | - | - | - | - |
| H3N2-4 | + | + | + | + | - | - | - | - |
| H3N2-5 | + | + | + | + | - | - | - | - |
| H5N1-1 | + | + | + | + | - | - | - | - |
| H5N1-2 | + | + | + | + | - | - | - | - |
| H5N1-3 | + | + | + | + | - | - | - | - |
| H5N1-4 | + | + | + | + | - | - | - | - |
| H5N1-5 | + | + | + | + | - | - | - | - |
| H7N9-1 | + | + | + | + | - | - | - | - |
| H7N9-2 | + | + | + | + | - | - | - | - |
| H7N9-3 | + | + | + | + | - | - | - | - |
| H7N9-4 | + | + | + | + | - | - | - | - |
| H7N9-5 | + | + | + | + | - | - | - | - |
| Yamagata-1 | + | + | + | + | - | - | - | - |
| Yamagata-2 | + | + | + | + | - | - | - | - |
| Yamagata-3 | + | + | + | + | - | - | - | - |
| Yamagata-4 | + | + | + | + | - | - | - | - |
| Yamagata-5 | + | + | + | + | - | - | - | - |
| Victoria-1 | + | + | + | + | - | - | - | - |
| Victoria-2 | + | + | + | + | - | - | - | - |
| Victoria-3 | + | + | + | + | - | - | - | - |
| Victoria-4 | + | + | + | + | - | - | - | - |
| Victoria-5 | + | + | + | + | - | - | - | - |
| RSV-G-1 | + | + | + | + | - | - | - | - |
| RSV-G-2 | + | + | + | + | - | - | - | - |
| RSV-G-3 | + | + | + | + | - | - | - | - |
| RSV-G-4 | + | + | + | + | - | - | - | - |
| RSV-G-5 | + | + | + | + | - | - | - | - |
| RSV-M-1 | + | + | + | + | - | - | - | - |
| RSV-M-2 | + | + | + | + | - | - | - | - |
| RSV-M-3 | + | + | + | + | - | - | - | - |
| RSV-M-4 | + | + | + | + | - | - | - | - |
| RSV-M-5 | + | + | + | + | - | - | - | - |
| RhV-A-1 | + | + | + | + | - | - | - | - |
| RhV-A-2 | + | + | + | + | - | - | - | - |
| RhV-A-3 | + | + | + | + | - | - | - | - |
| RhV-A-4 | + | + | + | + | - | - | - | - |
| RhV-A-5 | + | + | + | + | - | - | - | - |
| RhV-B-1 | + | + | + | + | - | - | - | - |
| RhV-B-2 | + | + | + | + | - | - | - | - |
| RhV-B-3 | + | + | + | + | - | - | - | - |
| RhV-B-4 | + | + | + | + | - | - | - | - |
| RhV-B-5 | + | + | + | + | - | - | - | - |
| RhV-C-1 | + | + | + | + | - | - | - | - |
| RhV-C-2 | + | + | + | + | - | - | - | - |
| RhV-C-3 | + | + | + | + | - | - | - | - |
| RhV-C-4 | + | + | + | + | - | - | - | - |
| RhV-C-5 | + | + | + | + | - | - | - | - |
| Adenovirus-1-1 | + | + | + | + | - | - | - | - |
| Adenovirus-1-2 | + | + | + | + | - | - | - | - |
| Adenovirus-1-3 | + | + | + | + | - | - | - | - |
| Adenovirus-1-4 | + | + | + | + | - | - | - | - |
| Adenovirus-1-5 | + | + | + | + | - | - | - | - |
| Adenovirus-2-1 | + | + | + | + | - | - | - | - |
| Adenovirus-2-2 | + | + | + | + | - | - | - | - |
| Adenovirus-2-3 | + | + | + | + | - | - | - | - |
| Adenovirus-2-4 | + | + | + | + | - | - | - | - |
| Adenovirus-2-5 | + | + | + | + | - | - | - | - |
| Adenovirus-3-1 | + | + | + | + | - | - | - | - |
| Adenovirus-3-2 | + | + | + | + | - | - | - | - |
| Adenovirus-3-3 | + | + | + | + | - | - | - | - |
| Adenovirus-3-4 | + | + | + | + | - | - | - | - |
| Adenovirus-3-5 | + | + | + | + | - | - | - | - |
| Adenovirus-4-1 | + | + | + | + | - | - | - | - |
| Adenovirus-4-2 | + | + | + | + | - | - | - | - |
| Adenovirus-4-3 | + | + | + | + | - | - | - | - |
| Adenovirus-4-4 | + | + | + | + | - | - | - | - |
| Adenovirus-4-5 | + | + | + | + | - | - | - | - |
| Adenovirus-5-1 | + | + | + | + | - | - | - | - |
| Adenovirus-5-2 | + | + | + | + | - | - | - | - |
| Adenovirus-5-3 | + | + | + | + | - | - | - | - |
| Adenovirus-5-4 | + | + | + | + | - | - | - | - |
| Adenovirus-5-5 | + | + | + | + | - | - | - | - |
| Adenovirus-7-1 | + | + | + | + | - | - | - | - |
| Adenovirus-7-2 | + | + | + | + | - | - | - | - |
| Adenovirus-7-3 | + | + | + | + | - | - | - | - |
| Adenovirus-7-4 | + | + | + | + | - | - | - | - |
| Adenovirus-7-5 | + | + | + | + | - | - | - | - |
| Adenovirus-55-1 | + | + | + | + | - | - | - | - |
| Adenovirus-55-2 | + | + | + | + | - | - | - | - |
| Adenovirus-55-3 | + | + | + | + | - | - | - | - |
| Adenovirus-55-4 | + | + | + | + | - | - | - | - |
| Adenovirus-55-5 | + | + | + | + | - | - | - | - |
| Enterovirus-A-1 | + | + | + | + | - | - | - | - |
| Enterovirus-A-2 | + | + | + | + | - | - | - | - |
| Enterovirus-A-3 | + | + | + | + | - | - | - | - |
| Enterovirus-A-4 | + | + | + | + | - | - | - | - |
| Enterovirus-A-5 | + | + | + | + | - | - | - | - |
| Enterovirus-B-1 | + | + | + | + | - | - | - | - |
| Enterovirus-B-2 | + | + | + | + | - | - | - | - |
| Enterovirus-B-3 | + | + | + | + | - | - | - | - |
| Enterovirus-B-4 | + | + | + | + | - | - | - | - |
| Enterovirus-B-5 | + | + | + | + | - | - | - | - |
| Enterovirus-C-1 | + | + | + | + | - | - | - | - |
| Enterovirus-C-2 | + | + | + | + | - | - | - | - |
| Enterovirus-C-3 | + | + | + | + | - | - | - | - |
| Enterovirus-C-4 | + | + | + | + | - | - | - | - |
| Enterovirus-C-5 | + | + | + | + | - | - | - | - |
| Enterovirus-D-1 | + | + | + | + | - | - | - | - |
| Enterovirus-D-2 | + | + | + | + | - | - | - | - |
| Enterovirus-D-3 | + | + | + | + | - | - | - | - |
| Enterovirus-D-4 | + | + | + | + | - | - | - | - |
| Enterovirus-D-5 | + | + | + | + | - | - | - | - |
| EB virus-1 | + | + | + | + | - | - | - | - |
| EB virus-2 | + | + | + | + | - | - | - | - |
| EB virus-3 | + | + | + | + | - | - | - | - |
| EB virus-4 | + | + | + | + | - | - | - | - |
| EB virus-5 | + | + | + | + | - | - | - | - |
| Measles-1 | + | + | + | + | - | - | - | - |
| Measles-2 | + | + | + | + | - | - | - | - |
| Measles-3 | + | + | + | + | - | - | - | - |
| Measles-4 | + | + | + | + | - | - | - | - |
| Measles-5 | + | + | + | + | - | - | - | - |
| CMV-1 | + | + | + | + | - | - | - | - |
| CMV-2 | + | + | + | + | - | - | - | - |
| CMV-3 | + | + | + | + | - | - | - | - |
| CMV-4 | + | + | + | + | - | - | - | - |
| CMV-5 | + | + | + | + | - | - | - | - |
| Rotavirus-1 | + | + | + | + | - | - | - | - |
| Rotavirus-2 | + | + | + | + | - | - | - | - |
| Rotavirus-3 | + | + | + | + | - | - | - | - |
| Rotavirus-4 | + | + | + | + | - | - | - | - |
| Rotavirus-5 | + | + | + | + | - | - | - | - |
| Norovirus-1 | + | + | + | + | - | - | - | - |
| Norovirus-2 | + | + | + | + | - | - | - | - |
| Norovirus-3 | + | + | + | + | - | - | - | - |
| Norovirus-4 | + | + | + | + | - | - | - | - |
| Norovirus-5 | + | + | + | + | - | - | - | - |
| Mumps-1 | + | + | + | + | - | - | - | - |
| Mumps-2 | + | + | + | + | - | - | - | - |
| Mumps-3 | + | + | + | + | - | - | - | - |
| Mumps-4 | + | + | + | + | - | - | - | - |
| Mumps-5 | + | + | + | + | - | - | - | - |
| VZV-1 | + | + | + | + | - | - | - | - |
| VZV-2 | + | + | + | + | - | - | - | - |
| VZV-3 | + | + | + | + | - | - | - | - |
| VZV-4 | + | + | + | + | - | - | - | - |
| VZV-5 | + | + | + | + | - | - | - | - |
| MP-1 | + | + | + | + | - | - | - | - |
| MP-2 | + | + | + | + | - | - | - | - |
| MP-3 | + | + | + | + | - | - | - | - |
| MP-4 | + | + | + | + | - | - | - | - |
| MP-5 | + | + | + | + | - | - | - | - |
| HKU1-1 | + | + | + | + | - | - | - | - |
| HKU1-2 | + | + | + | + | - | - | - | - |
| HKU1-3 | + | + | + | + | - | - | - | - |
| HKU1-4 | + | + | + | + | - | - | - | - |
| HKU1-5 | + | + | + | + | - | - | - | - |
| OC43-1 | + | + | + | + | - | - | - | - |
| OC43-2 | + | + | + | + | - | - | - | - |
| OC43-3 | + | + | + | + | - | - | - | - |
| OC43-4 | + | + | + | + | - | - | - | - |
| OC43-5 | + | + | + | + | - | - | - | - |
| NL63-1 | + | + | + | + | - | - | - | - |
| NL63-2 | + | + | + | + | - | - | - | - |
| NL63-3 | + | + | + | + | - | - | - | - |
| NL63-4 | + | + | + | + | - | - | - | - |
| NL63-5 | + | + | + | + | - | - | - | - |
| 229E-1 | + | + | + | + | - | - | - | - |
| 229E-2 | + | + | + | + | - | - | - | - |
| 229E-3 | + | + | + | + | - | - | - | - |
| 229E-4 | + | + | + | + | - | - | - | - |
| 229E-5 | + | + | + | + | - | - | - | - |
| hMPV-1 | + | + | + | + | - | - | - | - |
| hMPV-2 | + | + | + | + | - | - | - | - |
| hMPV-3 | + | + | + | + | - | - | - | - |
| hMPV-4 | + | + | + | + | - | - | - | - |
| hMPV-5 | + | + | + | + | - | - | - | - |
| Parainfluenza  virus-1-1 | + | + | + | + | - | - | - | - |
| Parainfluenza  virus-1-2 | + | + | + | + | - | - | - | - |
| Parainfluenza  virus-1-3 | + | + | + | + | - | - | - | - |
| Parainfluenza  virus-1-4 | + | + | + | + | - | - | - | - |
| Parainfluenza  virus-1-5 | + | + | + | + | - | - | - | - |
| Parainfluenza  virus-2-1 | + | + | + | + | - | - | - | - |
| Parainfluenza  virus-2-2 | + | + | + | + | - | - | - | - |
| Parainfluenza  virus-2-3 | + | + | + | + | - | - | - | - |
| Parainfluenza  virus-2-4 | + | + | + | + | - | - | - | - |
| Parainfluenza  virus-2-5 | + | + | + | + | - | - | - | - |
| Parainfluenza  virus-3-1 | + | + | + | + | - | - | - | - |
| Parainfluenza  virus-3-2 | + | + | + | + | - | - | - | - |
| Parainfluenza  virus-3-3 | + | + | + | + | - | - | - | - |
| Parainfluenza  virus-3-4 | + | + | + | + | - | - | - | - |
| Parainfluenza  virus-3-5 | + | + | + | + | - | - | - | - |
| Parainfluenza  virus-4-1 | + | + | + | + | - | - | - | - |
| Parainfluenza  virus-4-2 | + | + | + | + | - | - | - | - |
| Parainfluenza  virus-4-3 | + | + | + | + | - | - | - | - |
| Parainfluenza  virus-4-4 | + | + | + | + | - | - | - | - |
| Parainfluenza  virus-4-5 | + | + | + | + | - | - | - | - |
| Haemophilus  influenzae-1 | + | + | + | + | - | - | - | - |
| Haemophilus  influenzae-2 | + | + | + | + | - | - | - | - |
| Haemophilus  influenzae-3 | + | + | + | + | - | - | - | - |
| Haemophilus  influenzae-4 | + | + | + | + | - | - | - | - |
| Haemophilus  influenzae-5 | + | + | + | + | - | - | - | - |
| Streptococcus  pneumoniae-1 | + | + | + | + | - | - | - | - |
| Streptococcus  pneumoniae-2 | + | + | + | + | - | - | - | - |
| Streptococcus  pneumoniae-3 | + | + | + | + | - | - | - | - |
| Streptococcus  pneumoniae-4 | + | + | + | + | - | - | - | - |
| Streptococcus  pneumoniae-5 | + | + | + | + | - | - | - | - |
| Streptococcus  pyogenes-1 | + | + | + | + | - | - | - | - |
| Streptococcus  pyogenes-2 | + | + | + | + | - | - | - | - |
| Streptococcus  pyogenes-3 | + | + | + | + | - | - | - | - |
| Streptococcus  pyogenes-4 | + | + | + | + | - | - | - | - |
| Streptococcus  pyogenes-5 | + | + | + | + | - | - | - | - |
| Candida albicans-1 | + | + | + | + | - | - | - | - |
| Candida albicans-2 | + | + | + | + | - | - | - | - |
| Candida albicans-3 | + | + | + | + | - | - | - | - |
| Candida albicans-4 | + | + | + | + | - | - | - | - |
| Candida albicans-5 | + | + | + | + | - | - | - | - |
| Bordetella pertussis-1 | + | + | + | + | - | - | - | - |
| Bordetella pertussis-2 | + | + | + | + | - | - | - | - |
| Bordetella pertussis-3 | + | + | + | + | - | - | - | - |
| Bordetella pertussis-4 | + | + | + | + | - | - | - | - |
| Bordetella pertussis-5 | + | + | + | + | - | - | - | - |
| Mycoplasma  pneumoniae-1 | + | + | + | + | - | - | - | - |
| Mycoplasma  pneumoniae-2 | + | + | + | + | - | - | - | - |
| Mycoplasma  pneumoniae-3 | + | + | + | + | - | - | - | - |
| Mycoplasma  pneumoniae-4 | + | + | + | + | - | - | - | - |
| Mycoplasma  pneumoniae-5 | + | + | + | + | - | - | - | - |
| Chlamydia  pneumoniae-1 | + | + | + | + | - | - | - | - |
| Chlamydia  pneumoniae-2 | + | + | + | + | - | - | - | - |
| Chlamydia  pneumoniae-3 | + | + | + | + | - | - | - | - |
| Chlamydia  pneumoniae-4 | + | + | + | + | - | - | - | - |
| Chlamydia  pneumoniae-5 | + | + | + | + | - | - | - | - |
| Mycobacterium  tuberculosis-1 | + | + | + | + | - | - | - | - |
| Mycobacterium  tuberculosis-2 | + | + | + | + | - | - | - | - |
| Mycobacterium  tuberculosis-3 | + | + | + | + | - | - | - | - |
| Mycobacterium  tuberculosis-4 | + | + | + | + | - | - | - | - |
| Mycobacterium  tuberculosis-5 | + | + | + | + | - | - | - | - |
| PJP-1 | + | + | + | + | - | - | - | - |
| PJP-2 | + | + | + | + | - | - | - | - |
| PJP-3 | + | + | + | + | - | - | - | - |
| PJP-4 | + | + | + | + | - | - | - | - |
| PJP-5 | + | + | + | + | - | - | - | - |

As shown in Table S1, S2 and S3, 659 samples (230 positive and 429 negative samples) were tested using LFIA and rRT-PCR tests at the same time in the Fourth People's Hospital of Nanning, Chongqing University Three Gorges Hospital, and Chongqing Public Health Medical Treatment Center. And only four samples were tested as false positives, and nine samples were tested as false negatives. The total coincidence rate of the three hospitals was more than 95%.

Table S2 Chongqing Public Health Medical Treatment Center

| Test results （LFIA） | PCR results | | Total |
| --- | --- | --- | --- |
|  | Positive | Negative |  |
| Positive | 111 | 4 | 115 |
| Negative | 5 | 324 | 329 |
| Total | 116 | 328 | 444 |

Table S3 the Fourth People's Hospital of Nanning

| Test results （LFIA） | PCR results | | Total |
| --- | --- | --- | --- |
|  | Positive | Negative |  |
| Positive | 48 | 0 | 48 |
| Negative | 2 | 101 | 103 |
| Total | 50 | 101 | 151 |

Table S4 Chongqing University Three Gorges Hospital

| Test results （LFIA） | PCR results | | Total |
| --- | --- | --- | --- |
|  | Positive | Negative |  |
| Positive | 62 | 0 | 62 |
| Negative | 2 | 0 | 2 |
| Total | 64 | 0 | 64 |

We have collated the data of 659 patients. As shown in Table S5, disease stage, age, gender, and clinical manifestations have only a slight influence on the diagnosis.

Table S5 Patient statistics table

|  |  | Negative | Positive | False negative | False positive |
| --- | --- | --- | --- | --- | --- |
| Age | 0-30 | 38 | 24 | 2 | 0 |
|  | 30-60 | 239 | 133 | 6 | 2 |
|  | 60-90 | 152 | 73 | 1 | 2 |
| Gender | Male | 212 | 125 | 3 | 1 |
|  | Female | 217 | 105 | 6 | 3 |
| Stage of disease | mild | 0 | 79 | 5 | 0 |
|  | Moderate | 0 | 101 | 2 | 0 |
|  | Severe | 0 | 50 | 2 | 0 |
| Clinical manifestations | PCR positive | 0 | 50 | 2 | 0 |
|  | Cough and fever | 0 | 106 | 3 | 0 |
|  | Physical discomfort | 0 | 74 | 4 | 0 |
