# Supplementary figures and images for: A rapid lateral flow immunoassay strip for detection of SARS‐CoV‐2 antigen using latex microspheres
Source: J Clin Lab Anal. 2021 Nov 6;35(12):e24091. doi: 10.1002/jcla.24091 (PMC8646881; doi:10.1002/jcla.24091)

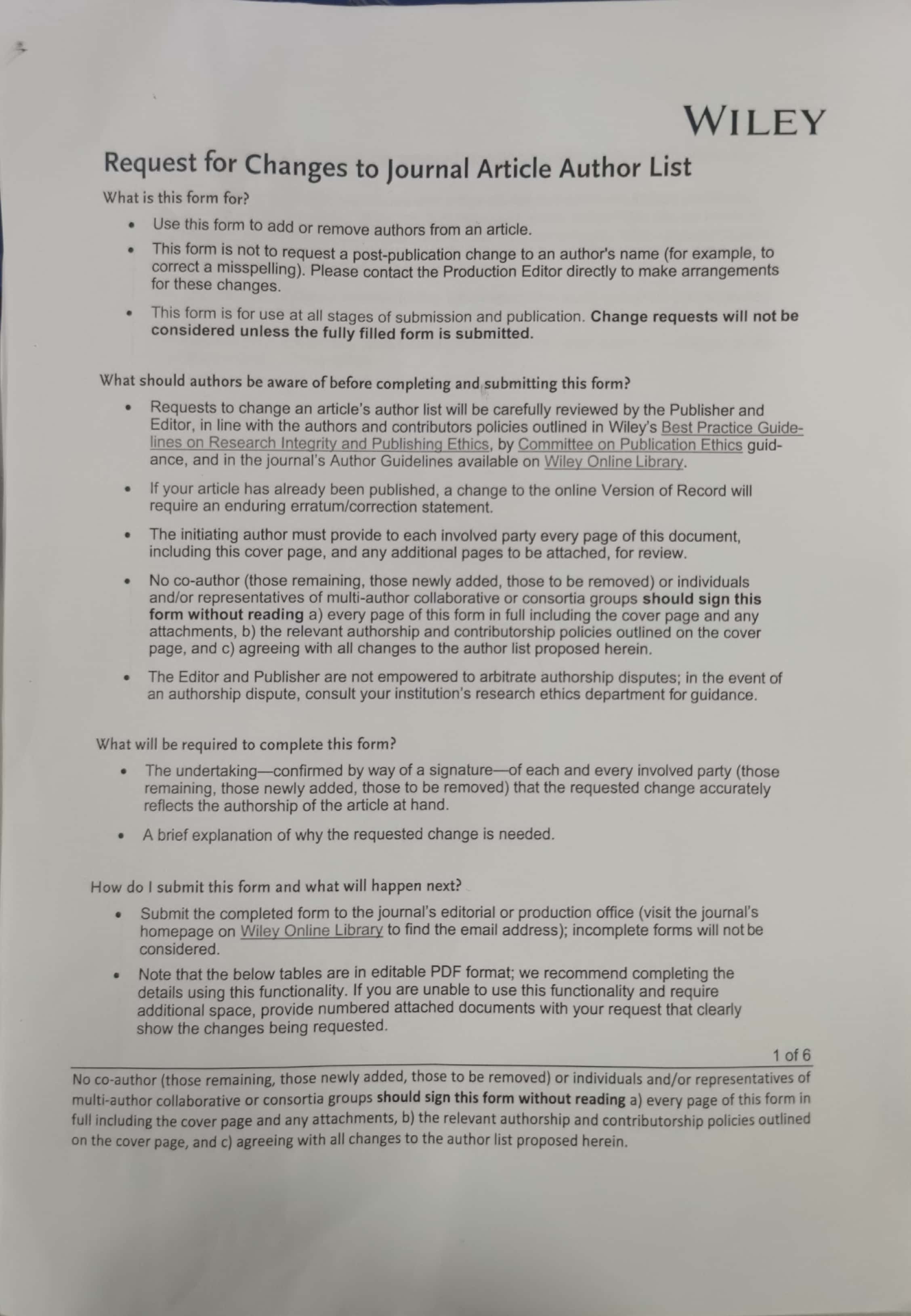


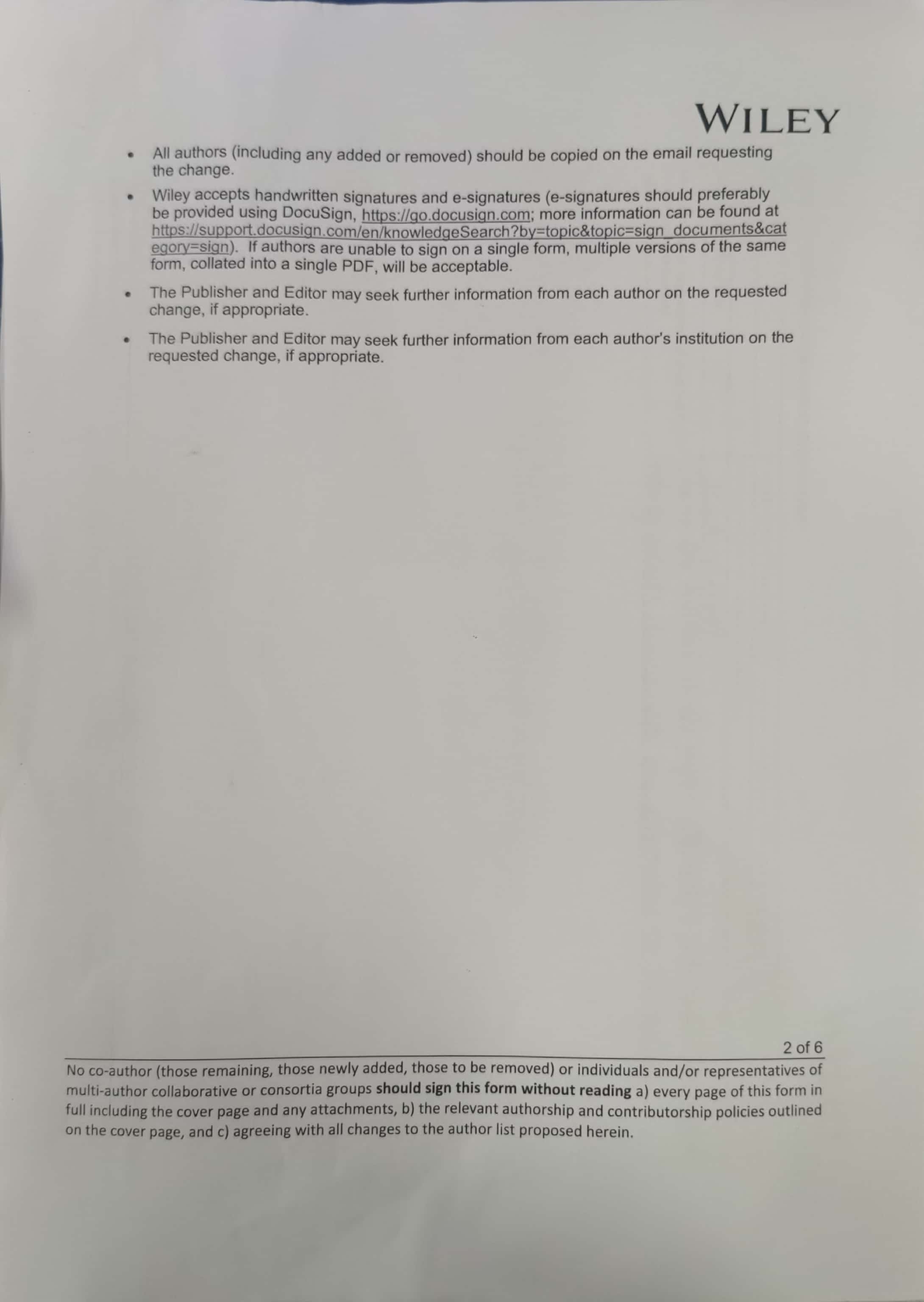


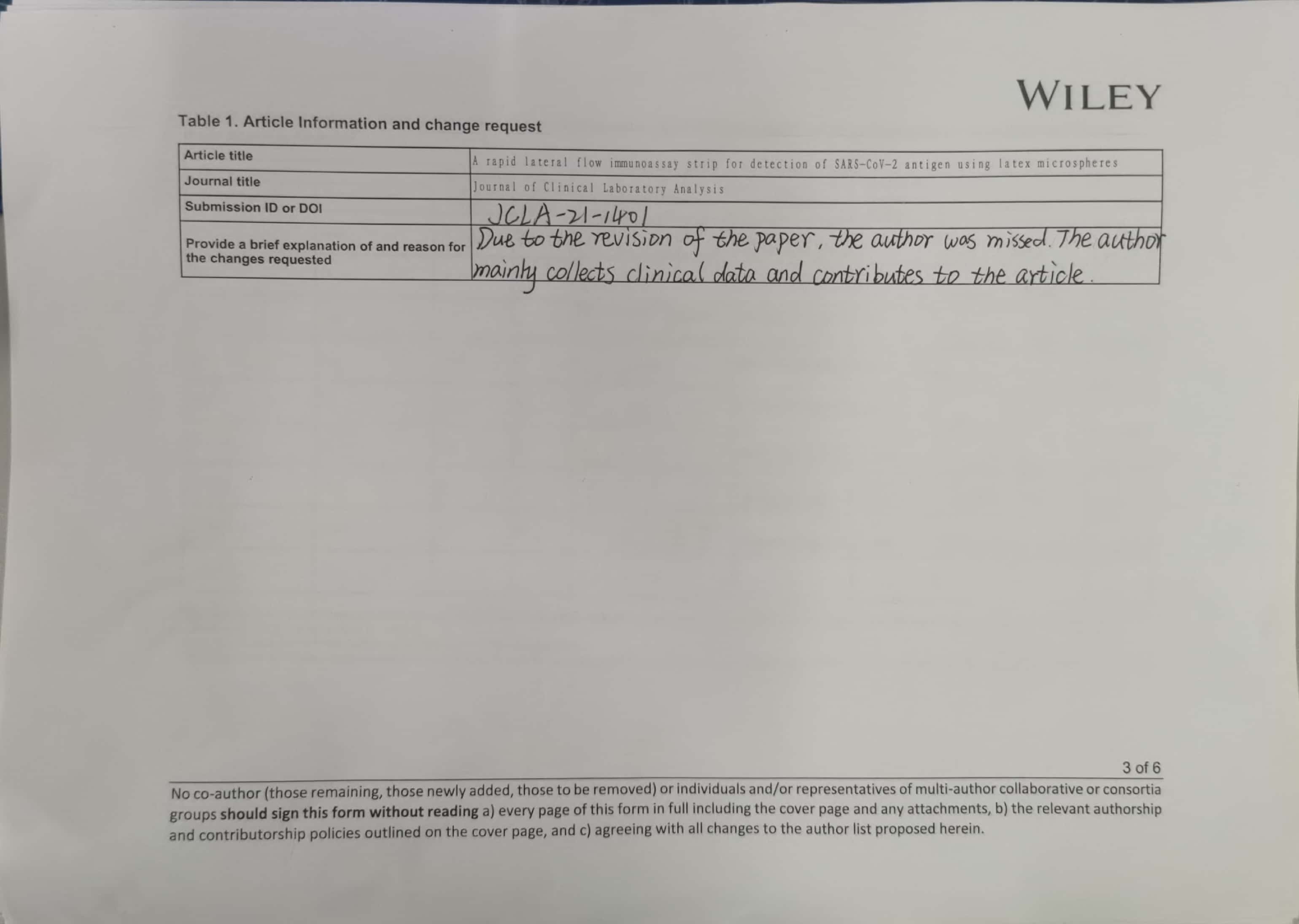


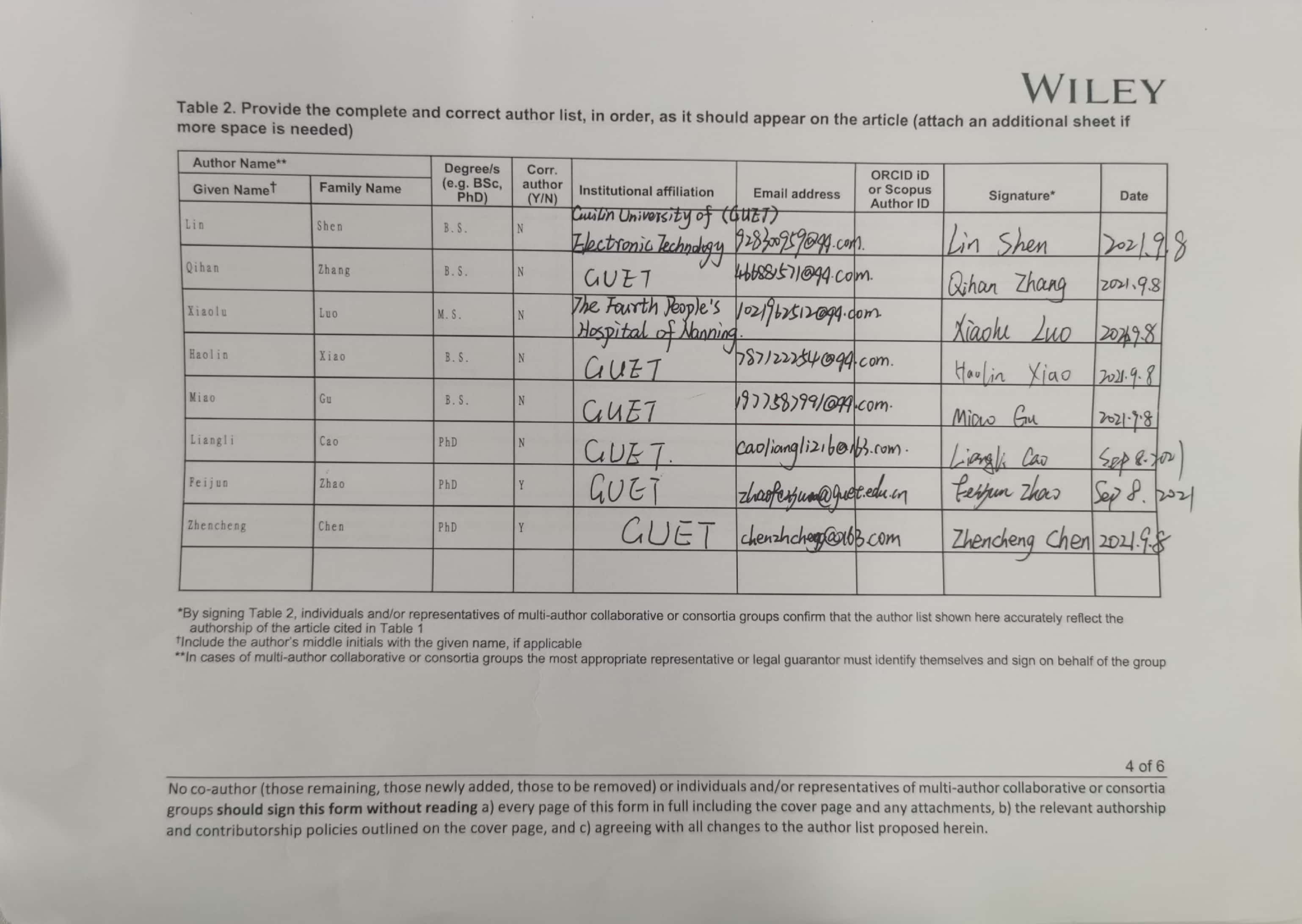


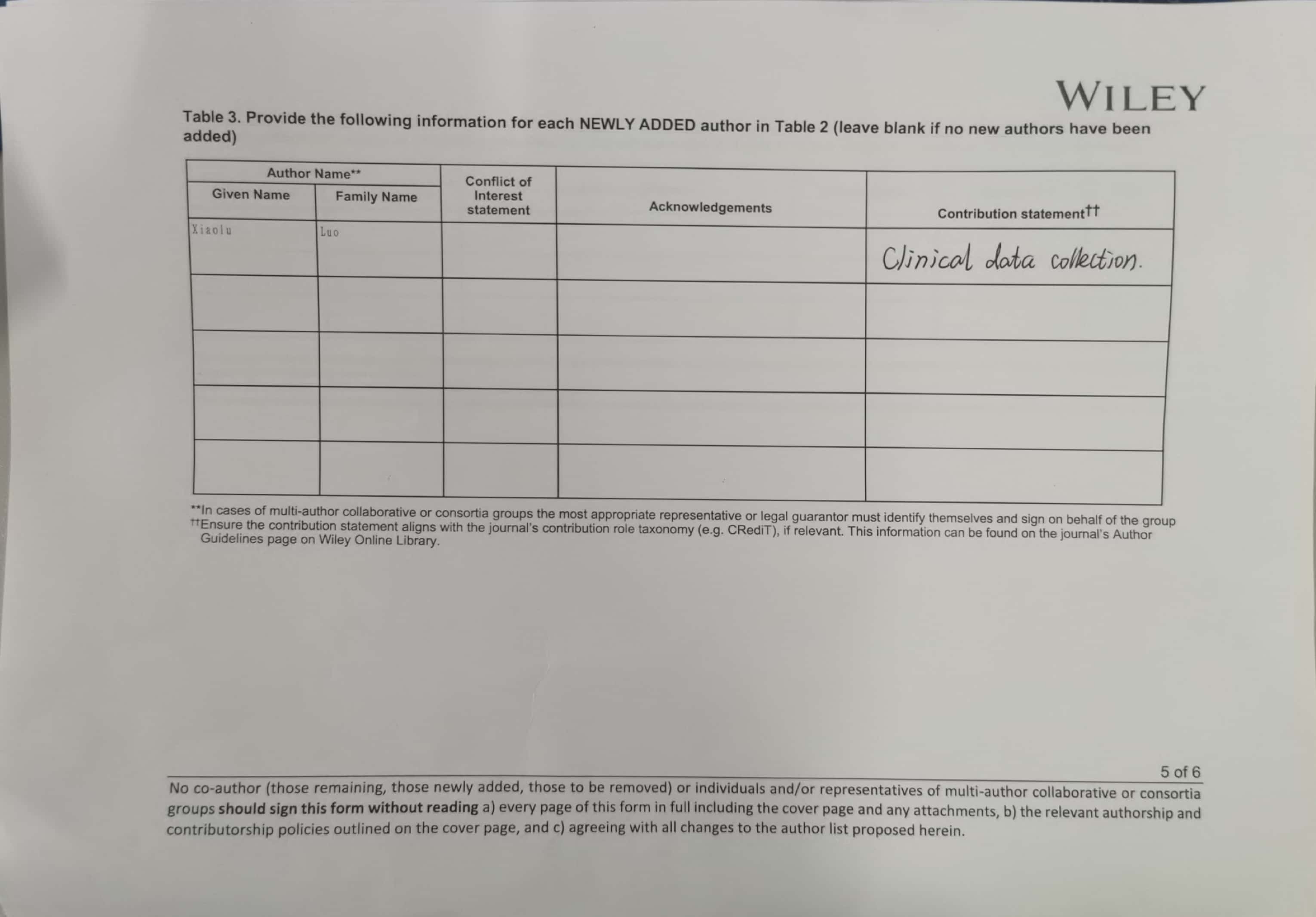


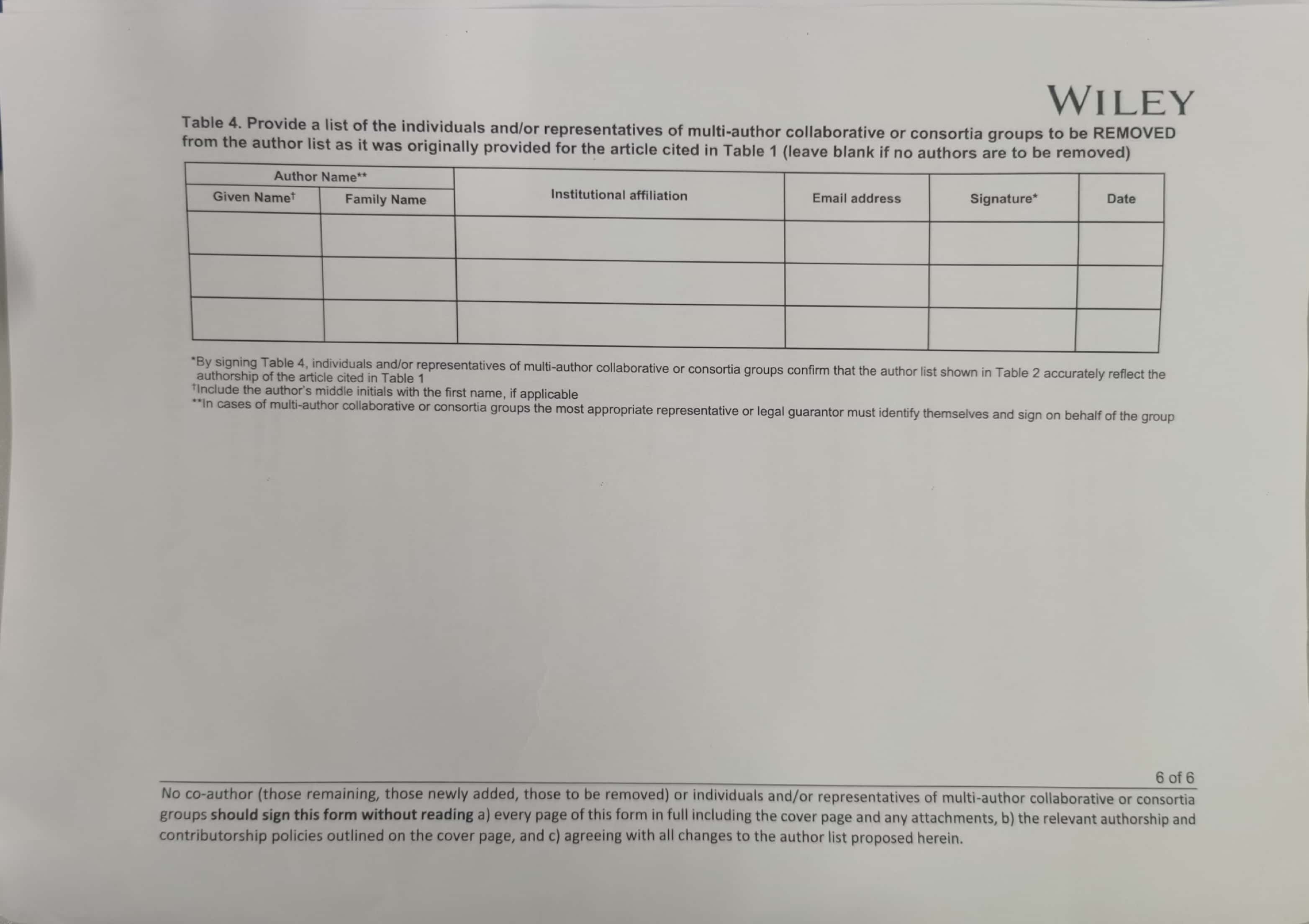

Supplement: Supplementary file 2 — Supplementary Material [file JCLA-35-e24091-s002.docx]
